# Thottapalayam virus is genetically distant to the rodent-borne hantaviruses, consistent with its isolation from the Asian house shrew (*Suncus murinus*)

**DOI:** 10.1186/1743-422X-4-80

**Published:** 2007-08-21

**Authors:** Pragya D Yadav, Martin J Vincent, Stuart T Nichol

**Affiliations:** 1 Special Pathogen Branch, Division of Viral and Rickettsial Diseases, National Center for Zoonotic, Vector-borne, and Enteric Diseases, Centers for Disease Control and Prevention, Atlanta, GA 30333, USA; 2 Microbial Containment Complex, National Institute of Virology, 130/1 Sus Road, Pashan, Pune 21, Maharashtra 411021, India

## Abstract

Thottapalayam (TPM) virus belongs to the genus *Hantavirus*, family *Bunyaviridae*. The genomes of hantaviruses consist of three negative-stranded RNA segments (S, M and L) encoding the virus nucleocapsid (N), glycoprotein (Gn, Gc), and polymerase (L) proteins, respectively. The genus *Hantavirus *contains predominantly rodent-borne viruses, with the prominent exception of TPM virus which was isolated in India in 1964 from an insectivore, *Suncus murinus*, commonly referred to as the Asian house shrew or brown musk shrew. Analysis of the available TPM virus S (1530 nt) RNA genome segment sequence and the newly derived M (3621 nt) and L (6581 nt) segment sequences demonstrate that the entire TPM virus genome is very unique. Remarkably high sequence differences are seen at the nucleotide (up to S – 47%, M – 49%, L – 38%) and protein (up to N – 54%, Gn/Gc – 57% and L – 39%) levels relative to the rodent-borne hantaviruses, consistent with TPM virus having a unique host association.

## Findings

Almost all hantaviruses (genus *Hantavirus*, family *Bunyaviridae*) are vectored by murid rodents of the *Murinae*, *Arvicolinae*, and *Sigmodontinae *subfamilies [1-3]. Hemorrhagic fever with renal syndrome (HFRS) is associated with infection by *Murinae*- and *Arvicolinae*-associated hantaviruses (e.g. Hantaan, Seoul and Puumala viruses) and hantavirus pulmonary syndrome (HPS) is associated with *Sigmodontinae*-associated hantaviruses (e.g. Sin Nombre and Andes viruses). Humans are infected by inhalation of aerosolized secreta or excreta from chronically infected rodents. As the molecular phylogeny of the rodent-borne hantaviruses largely mirrors the evolutionary history of their specific rodent hosts, these viruses are thought to have evolved over 10s of millions of years by co-speciation with their specific rodent hosts [1-4]. Despite being the first hantavirus isolated [5], Thottapalayam (TPM) virus remains something of an enigma, in that it was isolated not from a rodent, but from an insectivorous Asian house shrew or musk shrew (*Suncus murinus*) captured near Vellore, Tamil Nadu, India in1964. Later it was identified as a hantavirus based on electron microscopy morphology and cross-reactive serology [5-7]. However, TPM virus has to been shown to be the most antigenically distinct of all the hantaviruses [7,8]. In addition, the phylogenetic analysis of a small region of the S segment of TPM virus showed high divergence compared to other hantaviruses, suggestive of a unique reservoir host [9]. No further virus isolates have been obtained, and it remains unclear whether the Asian house shrew is the TPM virus primary reservoir or merely represents a spillover infection from some unidentified rodent host. To better characterize the virus and its relationship to other hantaviruses, a study to determine the complete genome of TPM virus was initiated.

TPM virus (strain VRC 66412) was grown in Vero E6 cells and harvested 12 days post-infection. Virus was inactivated in Tripure (Roche) and RNA isolated using the RNaid kit (Bio 101). The complete S segment sequence of TPM virus had been deposited in Genbank earlier by Song and colleagues (Genbank:AY526097). Alignment of the TPM virus S segment sequence with those of known hantaviruses allowed examination of conserved 3' and 5' RNA terminal sequences which could be used as the basis for PCR primer design to attempt to amplify the TPM virus M and L segment sequences. Following optimization of RT-PCR primers and reaction conditions, PCR products representing the entire TPM virus M (3621 bp) and L (6581 bp) RNA genome segments were successfully produced in single step RT-PCR reactions using the Superscript III single step RT-PCR system with Platinum Taq High fidelity (Invitrogen) according to the manufacturer's instructions. The newly designed primers included TPM-M-F1 (5'-TAGTAGTAGACTCCGCA-3) and TPM-M-R3684 (5'-TAGTAGTATRCTCCGCARG-3), and HANTA-L-F2, (5'-TAGTAGTAGACTCCGGAAG-3') and HANTA-L-R6577 (5'-TAGTAGTATGCTCCGRGAA-3') for M and L segment amplifications, respectively. The M segment RT reaction was performed at 50°C for 30 minute and PCR was performed at 94°C for 2 min, followed by 40 cycles of 94°C for 15 sec, 50°C for 30 sec, 68°C for 4 min, and a final extension at 68°C for 4 min. L segment reactions utilized identical conditions, except for 8 minute 68°C extension times. The amplified DNA products were separated on agarose gels, and recovered using Nucleotrap gel extraction kits (Clone Tech Lab). Cycle sequencing employed ABI Big-Dye 3.1 dye chemistry (Applied Biosystems, Foster City, CA) at 96°C -1 min, 96°C -10 sec, 45°C -5 sec and 60 °C -4 min for 25 cycles and resulting products were purified using Dyex 3.0 (Qiagen). Nucleotide sequence analysis via primer walking across these PCR products allowed the completion of the entire TPM virus genome sequence. Both DNA strands were sequenced and chromatogram data were assembled using Sequencher 4.1.4 software (Accelrys Inc.). Details of sequencing primers are available on request. The TPM virus complete M and L segment sequences have been made available [Genbank: DQ825770–DQ825771].

**Figure 1:**
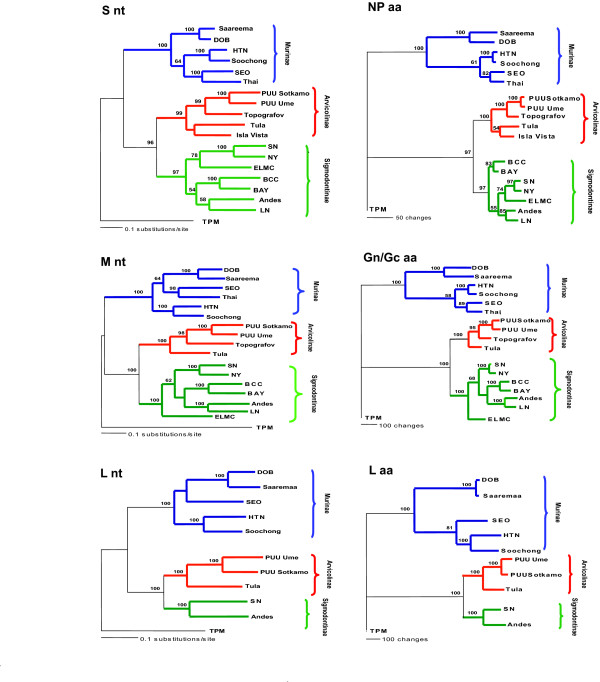
**Phylogenetic relationship of TPM virus relative to representatives of the rodent-borne hantaviruses**. Hantavirus sequences were aligned using the PILEUP program of the Wisconsin Package version 10.2 (Accelerys, Inc.) and phylogenetic analysis performed using PAUP 4.0b10 (Sinauer Association Inc., Sunderland, MA). Nucleotide sequences were analyzed by maximum likelihood method and maximum parsimony method was used for amino acids. Bootstrap confidence intervals were calculated using 500 heuristic search replicates. S segment sequence sources are: Hantaan (HTN) virus 76–118 M14626, Bayou (BAY) L36929, Black Creek Canal (BCC) virus l39949, Laguna Negra (LN) virus AF005727, Sin Nombre (SN) virus NM H10 L25784, New York (NY) virus RI-1 U09488, EI Moro Canyon (ELMC) virus RM97 U11427, Tula/Moravia/5302/95 Z69991, Puumala (PUU) virus Sotkamo X161036, PUU virus/Umea/hu NC_005224, Isla Vista (ISLA) virus U31534, Saaremaa virus 160V AJ009773, Dobrava (DOB) virus Ano-Poroia/Afl9/1999 AJ410615, Soochong virus SC-1 AY675349, Seoul (SEO) virus 80–39 AY273791, Hantavirus Thailand 741 AB288299, Topografov AJ011646, Andes virus CHI-7913 AY228237 and Thottapalayam (TPM) virus AY526097. M segment sources are: HTN virus 76–118, Bayou (BAY) L36930, BCC virus l39950, LN virus AF005728, SN virus NM H10 L25783, NY virus RI-1 U36801, ELMC virus RM97 U11428, Tula/Moravia/5302/95 Z69993, PUU virus Sotkamo X161034, PUU virus/Umea/hu NC_005223, Saaremaa virus160V AJ009774, DOB virus Ano-Poroia/Afl9/1999 AJ410616, Soochong virus SC-1 AY675353, SEO virus 80–39 S47716, Thailand 749 L08756, Topografov AJ011647, Andes virus CHI-7913 AY228238 and TPM virus. L segment sources are: HTN virus 76–118 X55901, SN virus NM H10 L37901, Tula/Moravia/5302/95 NC_005226, Puumala Sotkamo NC_005225, Puumala virus/Umea/hu AY526217, Saaremaa virus 160V AJ410618, DOB virus Ano-Poroia/Afl9/1999 AJ410617, Soochong virus SC-1 DQ056292, SEO virus 80–39 NC_005238, Andes virus CHI-7913 AY228239 and TPM virus.

The successful completion of the TPM virus genome sequence allowed comparison with the genomes of the rodent-borne hantaviruses, and demonstrated that TPM virus is the most genetically unique of all of the previously characterized hantaviruses. The TPM virus RNA segment nucleotide sequences differ from those of the other hantaviruses by 44.2–47.1 %, 46.8–49.2 %, and 37.0–38.0 % for the S, M and L segments, respectively. Deduced amino acid divergence was also very high, with 50.8–54.5 %, 54.9–57.2 %, and 37.6–38.9% identity differences found for the N, Gn/Gc and L proteins, respectively. Interestingly, TPM virus appears to be equally distant from the three main groups of hantaviruses associated with murid *Murinae*, *Arvicolinae *and *Sigmodontinae *subfamilies (Fig. 1). Despite the high differences observed, TPM virus displays many of the features common in the rodent-borne hantaviruses. For instance the S, M and L RNA segment lengths of 1530, 3621 and 6581 nucleotides, respectively, and the size of the ORFs and encoded proteins are all typical of those seen for the other hantaviruses.

Following the N ORF, the TPM virus S segment contains a highly variable long non-coding region similar to that seen in many hantaviruses, although the sequence diversity and length variation is such that these regions cannot be accurately aligned relative to the other hantaviruses. In terms of coding region, the N protein central region is highly conserved. This region has been shown to contain the RNA binding domain, which in the case of HTN virus has been mapped to a minimal region spanning amino acids 194–204 [10]. Studies of several hantaviruses have implicated the amino- and carboxy-termini in N protein interactions, and although the N protein amino-terminus of TPM virus is highly divergent relative to that of the other hantavirues, it is still predicted to form an anti-parallel coiled coil structure which is thought to be important in triggering N protein trimerization [11-13].

The M genome segment 3365 nucleotide long single ORF (position 40–3405) is predicted to encode a glycoprotein precursor of approximately 126 kDa. The overall structure appears similar to that of other hantaviruses, with approximate alignment of hydrophobic domains corresponding to signal peptide, and Gn and Gc transmembrane domains (data not shown). While most hantavirus glycoproteins have 5–7 predicted N-glycosylations sites, four potential N-glycosylation sites are conserved among all hantaviruses, three in Gn (e.g. amino acids 142, 357 and 409 in PUUV) and one in Gc (937 in PUUV) [14]. The TPM virus glycoprotein is predicted to contain 6 potential N- linked glycosylation sites, five sites in Gn and one in Gc, at aa positions 134, 289, 388, 505, 585, and 916. In general, the N glycosylation sites are highly conserved among hantaviruses and seem to be crucial for the conformation and function of the proteins which includes the proper transport, receptor binding and antigenicity [15]. The TPM virus glycoprotein also contains the previously identified WAASA amino acid motif (aa 633–637) which is conserved in all the rodent-borne hantaviruses and represents the cleavage signal for the processing of the mature Gn and Gc proteins [16]. In addition, a CPYC motif is found in TPM virus Gn carboxy region similar to that seen in the rodent-borne hantaviruses. Although the function of this motif is unclear for the hantaviruses, it has been shown with other viruses to be a redox site and play a role in cellular oxidation-reduction homeostasis [17].

Information on characterization of L protein of hantaviruses is scanty. A previous study identified five conserved motifs (motifs A, B, C, D and E) among all hantavirus RNA polymerases [18]. As expected, these motifs are conserved in the RNA polymerase of TPM virus.

Phylogenetic analysis of hantavirus S, M and L genome segment nucleotide and amino acid sequence differences clearly indicated similar branching patterns and topology and that hantavirus genomes are divided into 4 major phylogentic lineages which correspond to the viruses vectored by rodents in the subfamilies *Murinae*, *Sigmontinae *and *Arvicolinae *and the shrew-associated TPM virus (Fig. 1). Thus, the complete L and M genome data combined with previously published S segment data provide clear evidence that TPM virus is a very unique hantavirus in all its three segments and is not a recombinant which had acquired the S segment from an unknown ancestor.

TPM virus was isolated from an Asian house shrew (order Insectivora, family Soricidae, *Suncus murinus*), captured in Tamil Nadu, India [5]. The public health significance of the virus is currently unknown. A recent serosurvey in Tamil Nadu identified the presence of hantavirus IgM positivity in some human febrile illness cases [19,20]. In addition, anti-TPM virus antibodies were detected in sera from a febrile patient in Thailand and from two Asian house shrews captured in Indonesia [8]. However, no virus or sequences were obtained from these specimens, so while these data point towards the presence hantaviruses in India, Thailand and Indonesia, it is unclear whether these represent TPM virus or other hantavirus infections. The determination of the entire genome sequence of TPM virus will allow development of sensitive and specific molecular detection assays for use in screening cases of acute febrile illness in Asia, and insectivore investigations to provide insight into the public health significance and distribution of TPM virus.

Finally, it was recently reported that a novel hantavirus was detected in the Therese shrew (order Insectivora, family Soricidae, *Crocidura theresae*) captured in Guinea in western Africa [21]. In addition, new hantaviruses are reported to have been detected in 4 other shrew species in the family Soricidae from Eurasia and the Americas [22]. Complete genome sequences of these viruses will provide insight into the long evolutionary history of the hantaviruses, and the development of specific diagnostic assays should provide the means to assess the potential public health importance of these shrew-associated hantaviruses.

## Competing interests

The author(s) declare that they have no competing interests.

## Authors' contributions

PDY participated in virus growth and RNA purification, design, optimization and execution of RT-PCR reactions, sequence analysis, phylogenetic analysis, and preparation of the manuscript. MJV participated in design of RT-PCR primers and experiments and preparation of the manuscript. STN conceived of the study, participated in the design and coordination of the experiments and preparation of the manuscript.
